# One-step synthesis of platinum nanoparticles loaded in alginate bubbles

**DOI:** 10.1186/1556-276X-9-277

**Published:** 2014-05-30

**Authors:** Chih-Hui Yang, Wei-Ting Wang, Alexandru Mihai Grumezescu, Keng-Shiang Huang, Yung-Sheng Lin

**Affiliations:** 1 Department of Biological Science and Technology, I-Shou University, Kaohsiung 82445, Taiwan; 2 The School of Chinese Medicine for Post-Baccalaureate, I-Shou University, Kaohsiung 82445, Taiwan; 3 Department of Science and Engineering of Oxide Materials and Nanomaterials, Faculty of Applied Chemistry and Materials Science, University Politehnica of Bucharest, Bucharest 060042, Romania; 4 Department of Applied Cosmetology and Master Program of Cosmetic Science, Hungkuang University, Taichung 43302, Taiwan

**Keywords:** Platinum, Bubbles, Alginate, Nanoparticle

## Abstract

Composite particles with multifunctions have been extensively utilized for various applications. Bubble particles can be applied for ultrasound-mediated imaging, drug delivery, absorbers, cell culture, etc. This study proposes a one-step strategy to obtain Pt nanoparticles loaded in alginate bubbles. A needle-based droplet formation was used to generate uniform alginate particles about 2 mm in diameter. The hydrolysis reaction of NaBH_4_ was utilized to produce gaseous hydrogen and then trapped within alginate particles to form bubbles. The Pt^4+^ mixed with alginate solution was dropped into the reservoir to react with reducing NaBH_4_ and hardening CaCl_2_ to form Pt nanoparticles-alginate composite bubbles. Results indicate that the size of bubbles decreases with the CaCl_2_ concentration (1% ~ 20%), and size of bubbles increases with the NaBH_4_ concentration (1 ~ 20 mM). The advantages for the present approach include low cost, easy operation, and effective production of Pt nanoparticles-alginate composite bubbles.

## Background

The metal nanoparticles (NPs) are powerful products of nanotechnology, providing broad variety of applications in life science [1,2]. For example, drug delivery, cellular imaging, and biosensing have been extensively described [3-6]. The chemical versatility of metal NPs holds the potential to outclass in a number of applications [2]. These unique properties and applications of metal NPs are well reviewed [7-9]. Platinum is used in various applications such as catalysts in many organic reactions [10,11], preparation of organic dyes [12], and biomedical applications [13,14]. For example, the Pt NPs were employed for successful photothermal treatment of Neuro 2A cancer cell by using irradiation with 1,064 nm near-infrared pulse wave and the Nd YAG laser set at 3 W for 480 s. The Pt NPs increased 9°C in temperature leading to effective photothermal killing of cancer cells [15].

The Pt composite materials have gained much attention due to their good multifunctions [16,17]. Pt NPs-chitosan composite particles have been extensively studied over the last decade [18,19], and Pt NPs-chitosan composite bubbles are one of the most emerging and intriguing topics [20,21]. Bubble particles have import features entrapping air bubbles inside. Due to their low density, bubble particles can float on liquid surface for specific applications. They can also be applied as novel vehicles for ultrasound-mediated imaging and targeted drug delivery followed by burst release [22-27]. Besides, bubble particles can be utilized as absorbers to facilitate adsorption of substrates due to a high-surface area. Pt NPs-chitosan composite bubbles can be applied in controlled release and tissue engineering; however, chitosan carrier substrates will disintegrate and dissolve in acid solution such as gastric juices. Therefore, Pt NPs-chitosan composite bubbles are limited in acidic condition. Fortunately, alginate polymer provides a solution to overcome this problem. Alginate polymer has a dense structure to pass the acid solution. To our best knowledge, Pt NPs-alginate composite (Pt NPs@alginate) bubbles are seldom reported in literatures, and they can provide applications for wide pH ranges.

By extending our previous works to prepare uniform alginate particles [28-31] and alginate bubbles [32], this work further develops a novel one-step method to fabricate composite Pt NPs@alginate bubbles through a simple chemical reaction. The Pt NPs and bubbles within alginate particles are investigated and characterized. The manufactured alginate products will provide great promise for multifunctional applications.

## Methods

### Materials

Alginic acid sodium salt (Na-alginate, brown algae with viscosities 150 cp and 350 cp in 2% (*w*/*v*) solution at 25°C) and dihydrogen hexachloroplatinate (IV) hexahydrate, ACS, Premion, 99.95% were obtained from Alfa Aesar (Johnson Matthey Company, London, UK). Sodium borohydride (NaBH_4_) was purchased from Sigma (Sigma Chemical Co., St. Louis, MO, USA), and calcium chloride (CaCl_2_) was obtained from J.T. Baker (J.T. Baker Chemical Company, Phillipsburg, NJ, USA). All chemicals and solvents were of analytical reagent grade.

### Mechanism of bubbles formation

The gas source is from the hydrolysis of NaBH_4_ as following reaction [33]:

$$\mathrm{NaB}{\text{H}}_{4}+2\;{\text{H}}_{2}\text{O}\to \mathrm{NaB}{\text{O}}_{2}+4{\text{H}}_{2}.$$

The NaBH_4_ hydrolysis is spontaneous, and gaseous H_2_ generation continues with the hydrolysis reaction. Due to the density difference, generated H_2_ bubbles move upwards in the reservoir solution. After a dropwise addition of Na-alginate solution into the reservoir, gas bubbles were entrapped within alginate particles to be alginate bubbles. One alginate particle can hold many numbers of bubbles by random. After 30 min, alginate bubbles were collected by filter, washed twice with 30 mL dd-H_2_O, and finally collected and characterized.

### Preparation of Pt NPs@alginate bubbles

**Figure 1:**
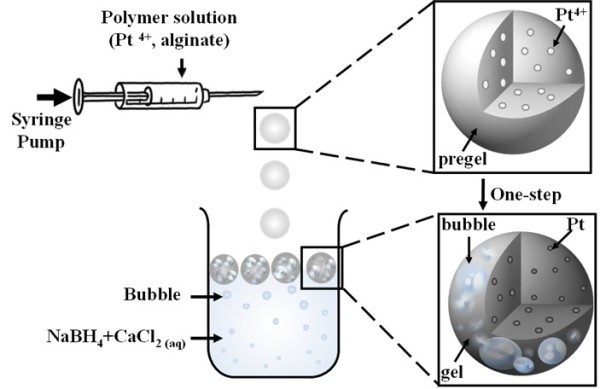
Schematic drawings of experimental setup.

Na-alginate (0.08 g dissolved in 2 mL of deionized water) and 2 mM platinum salt solution from dihydrogen hexachloroplatinate hexahydrate were mixed homogenously to be Pt^4+^ mixed Na-alginate (Pt^4+^-Na-alginate) solution. As shown in Figure 1, Pt^4+^-Na-alginate solution loaded in the syringe (TERUMO® syringe, 3 mL; Terumo, Tokyo, Japan) was extruded from the needle tip by a KDS230 syringe pump (KD Scientific Inc., Holliston, MA, USA). Under a constant injection rate, Pt^4+^-Na-alginate solution was broken up to form a series of isolatable Pt^4+^-Na-alginate droplets at the tip of the needle. The liquid in the receiving collector was filled with CaCl_2_ and NaBH_4_ for crosslinking alginate (by CaCl_2_) and generating Pt NPs (by NaBH_4_) and bubbles (by NaBH_4_), respectively. Pt^4+^-Na-alginate droplets are gelled *in situ* to be Ca-alginate particles when contacting Ca^2+^ ions. The NaBH_4_ plays a dual functional. One is a reducing agent to reduce Pt^4+^ to be Pt NPs by a chemical reduction reaction. The other one is gaseous H_2_ generation by a hydrolysis reaction. When the Pt^4+^-Na-alginate droplets immersed in the receiving collector, the Pt NPs@alginate bubbles are generated.

### Characterization

An optical microscope system (TE2000U, Nikon, Lewisville, TX, USA) and a USB digital microscope (UPG621, UPMOST Technology Corp., Taipei, Taiwan) were utilized to observe the morphology of the collected particles. To minimize selection bias, a total of more than 50 individual particles were analyzed to ensure statistical representation. X-ray diffraction (XRD, D2 Phaser, Bruker AXS Gmbh, Germany) patterns were obtained at room temperature by using Cu K-α radiation (30 kV/10 mA) with a range of 2*θ* = 20° ~ 80°, and a scanning rate of 4° min^−1^. Laser Raman spectroscopy was obtained using a Renishaw Microscope Raman Spectrometer (Renishaw plc., Gloucestershire, UK) from 200 to 1,100 cm^−1^ at room temperature. The 785-nm line of the laser was used as the excitation source, with the capability of supplying 300 mW. The morphology of the particle composites was analyzed using a scanning electron microscope (SEM, S-3400, Hitachi Ltd, Tokyo, Japan) and a transmission electron microscope (TEM, FEI Tecnai G2 20 S-Twin; FEI Company, Hillsboro, OR, USA) equipped with a METEK (PV 97–56700 ME) X-ray energy dispersive spectrometer (METEK Meteorologische Messtechnik GmbH, Elmshorn, Germany).

### Cell viability test

The viability of the control and the treated cells were evaluated using 3-(4,5-cimethylthiazol-2-yl)-2,5-diphenyl tetrazolium bromide (MTT) assay with human breast adenocarcinoma MCF-7 cells (1 × 10^4^/well) seeded in a 96-well microtiter plate with a 100 μL culture medium treated with various amounts of Pt NPs@alginate bubbles. After 1 day exposure, a 200-μL MTT solution was added to react with the cells for 4 h. After removal of the medium, 100 μL DMSO was added and examined at 595 nm using a microplate reader (Multiskan Ascent, Thermo Electron Corporation, Vantaa, Finland). The control group in the untreated well was considered to be 100%.

## Results and discussion

### Pt NPs@alginate bubbles

Alginate is a kind of polysaccharide from marine brown algae. A variety of fundamental properties such as excellent biodegradability and biocompatibility make alginate a very attractive material for applications. Alginate has been applied in diverse areas [34-36] including serving biomedical materials for drug delivery and tissue engineering, and being adsorbent materials for elimination of heavy metals or organic pollutants [37]. Due to acid dissolution, conventional Pt NPs@chitosan bubbles have constraint applications for limited pH conditions. Therefore, it is needed to develop Pt NPs@alginate bubbles for wide pH applications.

**Figure 2:**
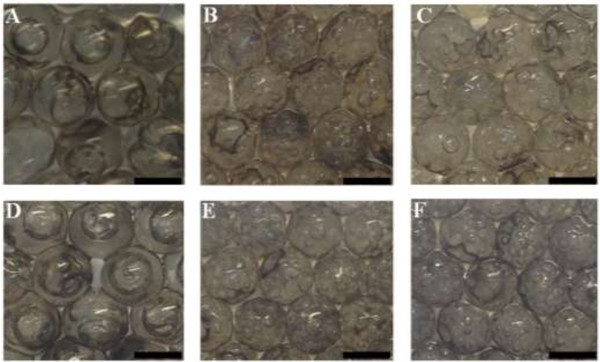
**Alginate bubbles with different CaCl**_**2 **_**concentrations. (A** and **D)** 1% CaCl_2_; **(B** and **E)** 10% CaCl_2_; **(C** and **F)** 20% CaCl_2_. Alginate in (**A** to **C**) and (**D** to **F**) are 150 and 350 cp, respectively. All scale bars are 2 mm.

Figure 2 shows the effects of CaCl_2_ concentration on Pt NPs@alginate bubbles. Results indicate that the size of the bubbles decreases with the CaCl_2_ concentration. The difference between the two alginate materials with distinct viscosities was not significant. The size of bubbles reaches 1 mm at 1% CaCl_2_, but only 0.4 mm at 20% CaCl_2._ The reason may be attributed to a lower crosslinking rate of alginate in a low CaCl_2_ concentration. The alginate pregel allows entrapped small bubbles merging into lager bubbles before gel network (solidification) formation in a low CaCl_2_ concentration.

**Figure 3:**
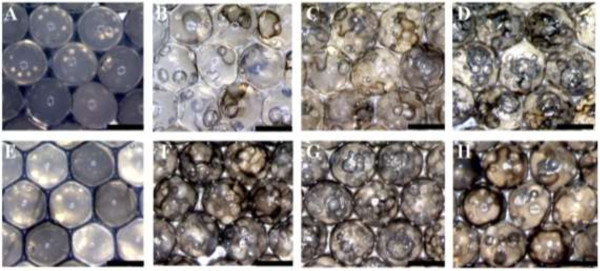
**Alginate bubbles with different NaBH**_**4 **_**concentrations. (A** and **E)** 1 mM NaBH_4_; **(B** and **F)** 5 mM NaBH_4_; **(C** and **G)** 10 mM NaBH_4_; **(D** and **H)** 20 mM NaBH_4_. Alginate in (**A** to **D**) and (**E** to **H**) are 150 and 350 cp, respectively. All scale bars are 2 mm.

Figure 3 shows the effects of NaBH_4_ concentration on Pt NPs@alginate bubbles. The results indicate that the number of bubbles within an alginate particle increases with NaBH_4_ concentration, but there is no significant difference between two alginate materials. There are no obvious bubbles in the low 1 mM NaBH_4_ due to the little amount of entrapped gas. Furthermore, the bubbles are well dispersed within the particle, but their size is not significantly varied with different NaBH_4_ concentrations. This phenomenon resulted from the high viscous alginate matrix to retard the fusion of bubbles.

Reduction reaction of Pt salts by reducing agents such as borohydrides and citrates is one of the convenient methods to prepare Pt NPs [38]. This study demonstrates a proof-of-concept approach for encapsulating the Pt NPs and bubbles into alginate particles utilizing simple reduction and hydrolysis reactions. Produced Pt NPs@alginate bubbles combined the characteristics of Pt NPs and bubbles. The composite bubble particles can provide wide applications, such as smart vehicles for ultrasound-mediated imaging and targeted drug delivery, and effective absorbers and catalysts for decomposing pollutants. In the future, this proposed strategy to formulate Pt NPs@alginate bubbles can also be applied for synthesis of other composite materials.

### Characterization

**Figure 4:**
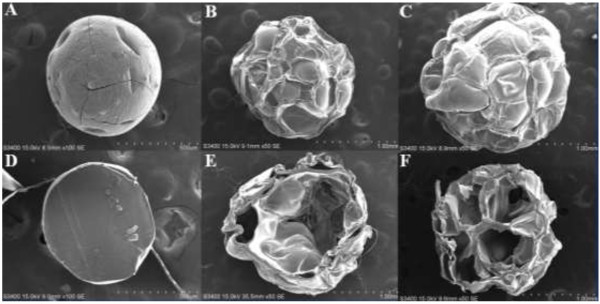
**SEM images of alginate bubbles with different NaBH**_**4 **_**concentrations.** Surface **(A** to **C)** and cross-section (**D** to **F**). (**A** and **D**) 0 mM NaBH_4_; (**B** and **E**) 10 mM NaBH_4_; (**C** and **F**) 20 mM NaBH_4_.

Figure 4 shows SEM images of Pt NPs@alginate bubbles. The exterior and interior morphologies of alginate particles obtained from different NaBH_4_ concentration are compared. In absence of NaBH_4_, there is no bubbles formation and the morphology is smooth and intact. For 10 and 20 mM NaBH_4_, ridges and cavities are found at particle surface and interior, showing entrapped bubbles.

**Figure 5:**
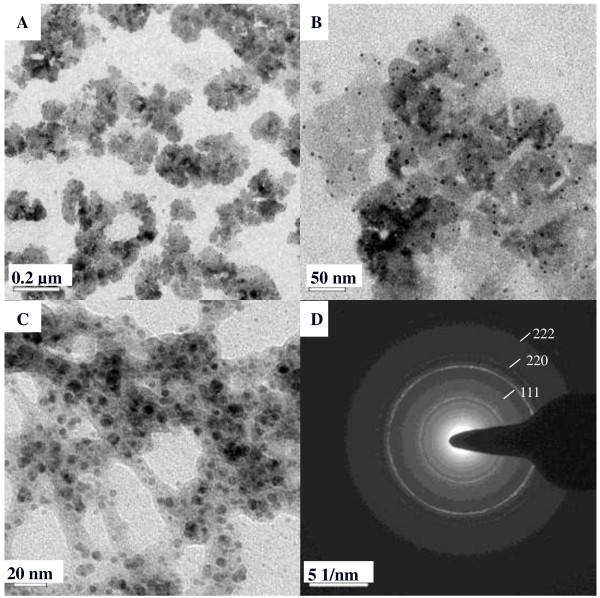
**TEM images and the electron diffraction pattern of Pt nanoparticles. (A-C).** TEM images of Pt nanoparticles with different magnifications. **(D)** Electron diffraction pattern of Pt nanoparticles.

**Figure 6:**
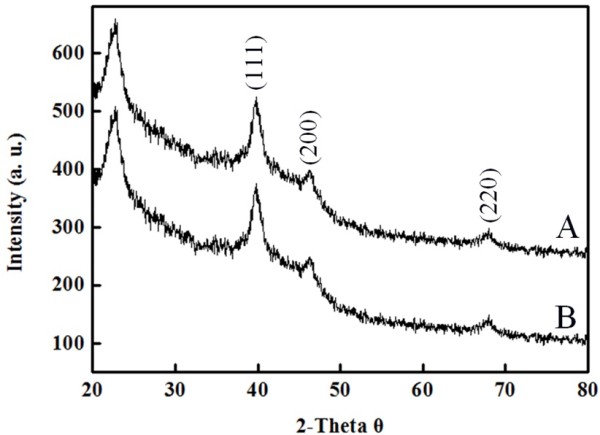
XRD patterns of Pt@alginate particles prepared from different alginate.

**Figure 7:**
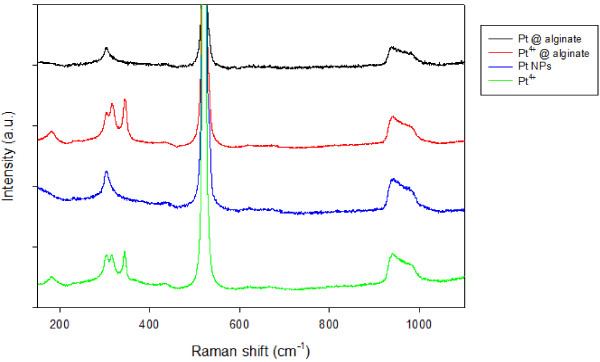
Raman patterns of different Pt compounds.

The TEM images shown in Figure 5 with different magnifications reveal that synthesized Pt NPs were nearly spherical and well dispersed in the Ca-alginate particle. The electron diffraction pattern of Pt NPs were indexed as (111), (220), and (222), indicating the polycrystalline characteristic. Figure 6 shows the XRD pattern of synthesized Pt NPs. Four distinct peaks at 39.6, 46.1, and 67.9 correspond to the crystal planes (111), (200), and (220) of cubic Pt NP structure, respectively. This result agrees with the finding in the electron diffraction data. Figure 7 is the Raman spectrum of different Pt substrates. There are different Raman patterns for Pt^4+^ and Pt. Compared to nonionic Pt, ionic Pt^4+^ shows more splits between 300 cm^−1^ and 350 cm^−1^. The Raman pattern of Pt NPs agrees with Pt NPs@alginate bubbles, and Pt^4+^ is consistent with Pt^4+^@alginate solution.

### Cytotoxicity

**Figure 8:**
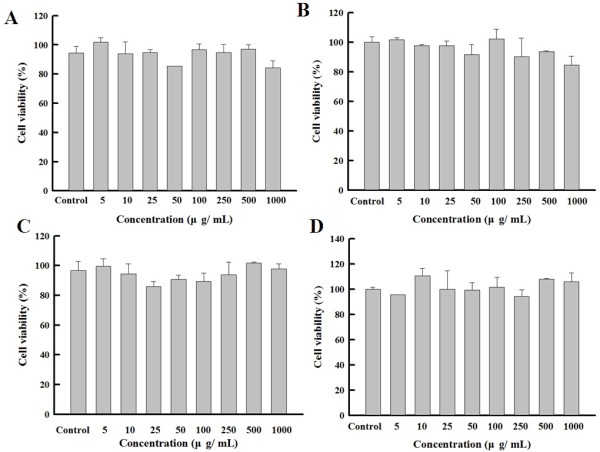
**Cytotoxicity induced by Pt@alginate bubbles on MCF-7 cells.** Alginate is 150 cp **(A** and **B)** and 350 cp **(C** and **D)**. The concentrations of CaCl_2_ are 10% (**A** and **C**) and 20% (**B** and **D**).

Figure 8 shows the cytotoxicity test of Pt NPs@alginate bubbles. The MCF-7 cells were used to test the cytotoxicity. Four kinds of alginate particles varying from alginate viscosity and CaCl_2_ concentration were tested. After a 24-h exposure to alginate particles ranging from 5 to 1,000 μg/mL, the cell viability was assayed. Results show that there was no significant difference among the control (without adding alginate particles) and the samples. Furthermore, the differences among the four kinds of alginate particles were rather indistinguishable. These results ensure the low cytotoxicity of prepared particles on the MCF-7 cells. Therefore, Pt NPs@alginate bubbles obtained in this study can be safely applied for biomedical applications in the future, such as the scaffold for cartilage tissue engineering [39].

### Particle morphology

Table 1 shows the particle morphology of chitosan and alginate materials in different pH conditions. The three particles, chitosan, alginate, and NPs@alginate bubbles, were compared along the immersion time. The results indicate that chitosan particles disintegrated in acid solution after 1 h immersion but the alginate material still had an entire particle shape. Although alginate displayed swelling in alkaline solution, the particles still remained. Therefore, NPs@alginate bubbles can provide more applications for wide pH ranges than conventional NPs@chitosan bubbles.

**Table 1:** Particle morphology of chitosan and alginate immersed in different solutions

**Material**	**Solution**	**Immersion time (hour)**			
		**0**	**0.5**	**1**	**2**
Chitosan	Gastric juice (pH 1.2)				
	PBS (pH 7.81)				
	Intestinal juice (pH 9.02)				
Alginate	Gastric juice (pH 1.2)				
	PBS (pH 7.81)				
	Intestinal juice (pH 9.02)				
Pt@alginate bubbles	Gastric juice (pH 1.2)				
	PBS (pH 7.81)				
	Intestinal juice (pH 9.02)				

## Conclusions

This paper developed a facile method to synthesize platinum nanoparticles within alginate bubbles. Sodium borohydrate was utilized to generate platinum NPs and gaseous hydrogen by reduction reaction and hydrolysis reaction, respectively. Bubbles entrapped within around 2-mm alginate particles increased with the borohydrate concentration and alginate viscosity. This proposed one-step method to prepare Pt NPs@alginate bubbles has advantages of low cost, easy operation, and effective pore formation. Compared with conventional Pt NPs@chitosan bubbles, Pt NPs@alginate bubbles provide more applications for wide pH ranges.

## Competing interests

The authors declare that they have no competing interest.

## Authors' contributions

CHY designed the study. WTW performed the entire search. AMG contributed to the discussion of the results. KSH and YSL wrote the manuscript and made the same contribution. All authors read and approved the final manuscript.

## References

[B1] Huang X Neretina S El-Sayed MA Gold nanorods: from synthesis and properties to biological and biomedical applications Adv Mater 2009 9 4880 4910 10.1002/adma.200802789 25378252

[B2] Matteini P Ratto F Rossi F Centi S Dei L Pini R Chitosan films doped with gold nanorods as laser‒activatable hybrid bioadhesives Adv. Mater 2010 9 4313 4316 10.1002/adma.201002228 20734385

[B3] Huang SL Liposomes in ultrasonic drug and gene delivery Adv Drug Deliver Rev 2008 9 1167 1176 10.1016/j.addr.2008.03.003 18479776

[B4] Kumar A Zhang X Liang XJ Gold nanoparticles: emerging paradigm for targeted drug delivery system Biotechnol Adv 2013 9 593 606 10.1016/j.biotechadv.2012.10.002 23111203

[B5] Yu F Zhang L Huang Y Sun K David AE Yang VC The magnetophoretic mobility and superparamagnetism of core-shell iron oxide nanoparticles with dual targeting and imaging functionality Biomaterials 2010 9 5842 5848 10.1016/j.biomaterials.2010.03.072 20434209 PMC2876197

[B6] Yoon H Jang J Conducting‒polymer nanomaterials for high‒performance sensor applications: issues and challenges Adv Funct Mater 2009 9 1567 1576 10.1002/adfm.200801141

[B7] Ando J Yano TA Fujita K Kawata S Metal nanoparticles for nano-imaging and nano-analysis Phys Chem Chem Phys 2013 9 13713 13722 10.1039/c3cp51806j 23861007

[B8] Pelgrift RY Friedman AJ Nanotechnology as a therapeutic tool to combat microbial resistance Adv Drug Deliv Rev 2013 9 1803 1815 10.1016/j.addr.2013.07.011 23892192

[B9] Tauran Y Brioude A Coleman AW Rhimi M Kim B Molecular recognition by gold, silver and copper nanoparticles World J Biol Chem 2013 9 35 63 23977421 10.4331/wjbc.v4.i3.35 PMC3746278

[B10] Bratlie KM Lee H Komvopoulos K Yang P Somorjai GA Platinum nanoparticle shape effects on benzene hydrogenation selectivity Nano Lett 2007 9 3097 3101 10.1021/nl0716000 17877408

[B11] Goor-Dar M Travitsky N Peled E Study of hydrogen redox reactions on platinum nanoparticles in concentrated HBr solutions J Power Sources 2012 9 111 115

[B12] Santhanalakshmi J Kasthuri J Rajendiran N Studies on the platinum and ruthenium nanoparticles catalysed reaction of aniline with 4-aminoantipyrine in aqueous and microheterogeneous media J Mol Catal A: Chem 2007 9 283 291 10.1016/j.molcata.2006.10.012

[B13] Bhattacharya R Mukherjee P Biological properties of “naked” metal nanoparticles Adv Drug Deliv Rev 2008 9 1289 1306 10.1016/j.addr.2008.03.013 18501989

[B14] Song JY Kwon EY Kim BS Biological synthesis of platinum nanoparticles using Diopyros kaki leaf extract Bioproc Biosyst Eng 2010 9 159 164 10.1007/s00449-009-0373-2 19701776

[B15] Manikandan M Hasan N Wu HF Platinum nanoparticles for the photothermal treatment of Neuro 2A cancer cells Biomaterials 2013 9 5833 5842 10.1016/j.biomaterials.2013.03.077 23642996

[B16] Chen S Fu P Yin B Yuan R Chai Y Xiang Y Immobilizing Pt nanoparticles and chitosan hybrid film on polyaniline naofibers membrane for an amperometric hydrogen peroxide biosensor Bioproc Biosyst Eng 2011 9 711 719 10.1007/s00449-011-0520-4 21318624

[B17] Ekrami-Kakhki MS Khorasani-Motlagh M Noroozifar M Platinum nanoparticles self-assembled onto chitosan membrane as anode for direct methanol fuel cell J Appl Electrochem 2011 9 527 534 10.1007/s10800-011-0273-4

[B18] Huang L Zhai M Peng J Xu L Li J Wei G Synthesis, size control and fluorescence studies of gold nanoparticles in carboxymethylated chitosan aqueous solutions J Colloid Interf Sci 2007 9 398 404 10.1016/j.jcis.2007.07.039 17707389

[B19] Wei D Ye Y Jia X Yuan C Qian W Chitosan as an active support for assembly of metal nanoparticles and application of the resultant bioconjugates in catalysis Carbohyd Res 2010 9 74 81 10.1016/j.carres.2009.10.008 19932470

[B20] Doshi N Mitragotri S Designer biomaterials for nanomedicine Adv Funct Mater 2009 9 3843 3854 10.1002/adfm.200901538

[B21] Cavalli R Bisazza A Trotta M Argenziano M Civra A Donalisio M Lembo D New chitosan nanobubbles for ultrasound-mediated gene delivery: preparation and in vitro characterization Int J Nanomed 2012 9 3309 3318 10.2147/IJN.S30912 PMC3396386 22802689

[B22] Dressaire E Bee R Bell DC Lips A Stone HA Interfacial polygonal nanopatterning of stable microbubbles Science 2008 9 1198 1201 10.1126/science.1154601 18511685

[B23] Capece S Chiessi E Cavalli R Giustetto P Grishenkov D Paradossi G A general strategy for obtaining biodegradable polymer shelled microbubbles as theranostic devices Chem Commun 2013 9 5763 5765 10.1039/c3cc42037j 23689681

[B24] Hosny NA Mohamedi G Rademeyer P Owen J Wu Y Tang MX Eckersley RJ Stride E Kuimova MK Mapping microbubble viscosity using fluorescence lifetime imaging of molecular rotors Proc Natl Acad Sci 2013 9 9225 9230 10.1073/pnas.1301479110 23690599 PMC3677502

[B25] Geers B De Wever O Demeester J Bracke M De Smedt SC Lentacker I Targeted liposome‒loaded microbubbles for cell‒specific ultrasound‒triggered drug delivery Small 2013 9 4027 4035 10.1002/smll.201300161 23737360

[B26] Noble ML Kuhr CS Graves SS Loeb KR Sun SS Keilman GW Morrison KP Paun M Storb RF Miao CH Ultrasound-targeted microbubble destruction-mediated gene delivery into canine livers Mol Ther 2013 9 1687 1694 10.1038/mt.2013.107 23732985 PMC3776626

[B27] Villa R Cerroni B Viganò L Margheritelli S Abolafio G Oddo L Paradossi G Zaffaroni N Targeted doxorubicin delivery by chitosan-galactosylated modified polymer microbubbles to hepatocarcinoma cells Colloids Surf B Biointerfaces 2013 9 434 442 23759384 10.1016/j.colsurfb.2013.04.022

[B28] Huang KS Yang CH Lin YS Wang CY Lu K Chang YF Wang YL Electrostatic droplets assisted synthesis of alginate microcapsules Drug Deliv Transl Res 2011 9 289 298 10.1007/s13346-011-0020-8 25788363

[B29] Huang KS Lin YS Yang CH Tsai CW Hsu MY In situ synthesis of twin monodispersed alginate microparticles Soft Matter 2011 9 6713 6718 10.1039/c0sm01361g

[B30] Wang CY Yang CH Lin YS Chen CH Huang KS Anti-inflammatory effect with high intensity focused ultrasound-mediated pulsatile delivery of diclofenac Biomaterials 2012 9 1547 1553 10.1016/j.biomaterials.2011.10.047 22082618

[B31] Lin YS Yang CH Hsu YY Hsieh CL Microfluidic synthesis of tail‒shaped alginate microparticles using slow sedimentation Electrophoresis 2013 9 425 431 10.1002/elps.201200282 23161405

[B32] Huang KS Lin YS Chang WR Wang YL Yang CH A facile fabrication of alginate microbubbles using a gas foaming reaction Molecules 2013 9 9594 9602 10.3390/molecules18089594 23941880 PMC6269812

[B33] Demirci UB Miele P Cobalt in NaBH_4_ hydrolysis Phys Chem Chem Phys 2010 9 14651 14665 10.1039/c0cp00295j 20944835

[B34] Coppi G Iannuccelli V Alginate/chitosan microparticles for tamoxifen delivery to the lymphatic system Int J Pharmaceut 2009 9 127 132 10.1016/j.ijpharm.2008.09.040 18940240

[B35] Chen CC Fang CL Al-Suwayeh SA Leu YL Fang JY Transdermal delivery of selegiline from alginate–pluronic composite thermogels Int J Pharmaceut 2011 9 119 128 10.1016/j.ijpharm.2011.05.060 21645593

[B36] Balaure PC Andronescu E Grumezescu AM Ficai A Huang KS Yang CH Chifiriuc CM Lin YS Fabrication, characterization and *in vitro* profile based interaction with eukaryotic and prokaryotic cells of alginate–chitosan–silica biocomposite Int J Pharmaceut 2013 9 555 561 10.1016/j.ijpharm.2012.10.045 23178215

[B37] Barbetta A Barigelli E Dentini M Porous alginate hydrogels: synthetic methods for tailoring the porous texture Biomacromolecules 2009 9 2328 2337 10.1021/bm900517q 19591464

[B38] Kumar KM Mandal BK Tamminaa SK Green synthesis of nano platinum using naturally occurring polyphenols RSC Adv 2013 9 4033 4039 10.1039/c3ra22959a

[B39] Wang CC Yang KC Lin KH Liu HC Lin FH A highly organized three-dimensional alginate scaffold for cartilage tissue engineering prepared by microfluidic technology Biomaterials 2011 9 7118 7126 10.1016/j.biomaterials.2011.06.018 21724248

